# The Role of Stimulus Uncertainty and Curiosity in Attention Control

**DOI:** 10.1027/1618-3169/a000608

**Published:** 2024-04-29

**Authors:** Seema Prasad, Bernhard Hommel

**Affiliations:** ^1^ Department of Child and Adolescent Psychiatry, Faculty of Medicine, TU Dresden, Germany; ^2^ School of Psychology, Shandong Normal University, Shandong, PR China

**Keywords:** curiosity, attention, cueing, uncertainty, motivation, capture

## Abstract

**Abstract:** Most cognitive psychological studies assume that participants in lab-based tasks maintain a single goal based on task instructions. However, people can be motivated by other factors, such as curiosity. We examined if people attend to seemingly task-irrelevant information out of curiosity by manipulating stimulus uncertainty in a cueing paradigm. Participants were presented with an abrupt-onset cue followed by a letter target (E or H). Next, a mask either at the target location (low uncertainty) or at all four locations (high uncertainty) was shown. We expected high uncertainty to induce a state of curiosity that in turn influences the processing of the cue. Cueing effects were greater in the high-uncertainty condition compared to the low-uncertainty condition. In Experiment 2, we additionally elicited self-report ratings on curiosity. In sum, target-specific uncertainty leads to greater processing of task-irrelevant peripheral cues across two experiments. We tentatively conclude that uncertainty modulates attention control and further research is necessary to examine if this is indeed due to curiosity induced by uncertainty.


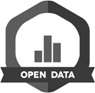


One of the main objectives of studying human attention has been to unearth the principles governing attentional selection: What do humans attend to and why? Existing models of attentional selection propose that what we pay attention to is decided by the goals of the individual (known as “top-down” or “goal-driven” selection) and/or by the salience of the information in the environment (known as “bottom-up” or “stimulus-driven” selection). For many years, this was the dominant view of most attention theorists. More recently, Awh et al. (2012) proposed a third factor that drives attention: selection history. In all such accounts, any behavior that is in contradiction to the explicitly stated (through instruction) goals of the task is considered a failure of goal-driven control. The implicit assumption underlying this conclusion is that, at any given point in time, participants can have only one goal which is the explicitly stated goal given to the participant during an experimental session (Hommel, 2022; Hommel & Wiers, 2017). However, “the subject does not hang up his nervous system on entering a laboratory and put on one of quite a different design” (Berlyne, 1960, p. 1). Thus, it is possible, and probably even likely, that individuals come into the laboratory with multiple goals. Some of these goals may be current and temporary, such as meeting friends right after the experiment, and some more chronic (Hommel, 2022). One of these chronic goals is curiosity – which is with us from birth (Bahrick et al., 1981; Hunter & Ames, 1988). If we assume that people will not switch off all these goals when entering a psychological laboratory, it seems reasonable to assume that they can contribute to attention control. In this study, we tested whether curiosity operationalized as uncertainty contributes to attention capture.

One of the paradigms used to study attention capture in a laboratory setting is the Posner cueing paradigm (Posner, 1980). In a standard Posner cueing task, peripheral cues are presented at two (or sometimes four) locations on the screen followed by a target at one of the cued locations (cued trials) or at a different location (uncued trials). Responses are typically faster to targets at cued locations compared to uncued locations, suggesting that peripheral cues capture attention and influence responses to the target despite being nonpredictive of the target location. This is taken as an example of bottom-up or stimulus-driven attentional selection because the peripheral cues capture attention although they are “task-irrelevant” and are not useful in finding the target. The implicit, often untested, assumption here is that to optimize performance on the instructed task goal (i.e., to search for the target) is the only relevant motivational factor for the participant during the experiment. This may not necessarily be true – what is typically considered task-irrelevant processing could be a manifestation of people’s curiosity toward uncertain visual displays.

Curiosity is defined as information-seeking behavior for its own sake and is driven by the need to reduce uncertainty in the environment (Berlyne, 1960; Kidd & Hayden, 2015; van Lieshout et al., 2020). Thus, an increase in uncertainty is associated with increase in curiosity which encourages people to explore their environment to reduce the uncertainty (Gottlieb, 2023; Gottlieb et al., 2020). In one of the earliest studies on curiosity, Nicki (1970) found that when presented with images of blurred objects, people chose to view the clearer image of the blurred object compared to a clearer image of a different object. This was taken to suggest that blurring makes people curious about the blurred object and they want to reduce this uncertainty by viewing the clearer image. Animals have also been shown to seek information that is not useful, or sometimes even costly to reduce uncertainty (Bromberg-Martin & Hikosaka, 2009; Wang & Hayden, 2019). People reported being more curious when the outcome of a lottery task is more uncertain, even when the outcome-related information does not help them perform better (Van Lieshout et al., 2018, 2021). Curiosity makes people seek information, not because it helps them achieve any other goal or reward, but because the information itself (i.e., the reduction in uncertainty) is considered a reward. Thus, it is possible to argue that increasing uncertainty would lead to greater attention being paid to information – in ways that may look like unintentional bottom-up distraction but that actually reflects the satisfaction of a top-down operating goal.

## Experiment 1

We wanted to examine if cueing effects observed in a Posner cueing task can be modulated by inducing uncertainty in the elements of the paradigm. Our key hypothesis was that cueing effects can be modulated by participants’ curiosity about the uncertainty in their visual environment. To examine this, we adopted the experimental design of Shiu and Pashler (1994) with some modifications. In Shiu and Pashler, a peripheral cue was presented in one of the four placeholders followed by a target digit (4, 5, 6, or 7). The cue location matched the target location on 75% of the trials. The target digit was followed by a single mask at the target location or four masks at all four locations. Cueing effects were observed only in the four-masks condition. The authors argued that cueing effects were a result of reduction in decision noise rather than signal enhancement as claimed by capacity-limitation models. In the single-mask condition, there was less decision noise as there were no other elements in the display that could be confused with the target. In the four-masks condition, however, the target letter could be confused with the masks in the four locations. In this case, the spatial cues help in excluding or weighting down unlikely target locations, thereby affecting performance.

We used this design to study the effect of uncertainty on attention control. Uncertainty regarding an event is usually greater either when the range of possibilities is greater or when the event has equal chance of materializing or not materializing (Berlyne, 1960). The four-masks condition represented high uncertainty regarding the target location as the target could be present in any of the four locations. The single-mask condition represented low uncertainty as the participants could know the location of the target with certainty. We hypothesized that the four-masks condition would induce a state of uncertainty, which would, in turn, influence the processing of the cue. It is important to note that the uncertainty here is associated with target location and not with the cues. This was intentional as we wanted to investigate noninstrumental information seeking, which is the definition of curiosity. In this experiment, the four masks made the target location uncertain. Therefore, any information seeking behavior we observe in this paradigm is likely related to curiosity.

We also included a third condition that was not present in Shiu and Pashler (1994). There was a block of single-mask trials with increased task difficulty. The trials in this block resembled the trials in the single-mask (easy task) block, except for the target display. The contrast of the target letter was reduced to increase the difficulty of the task (Snowden et al., 2001). This block was added to distinguish between the effects of increased task difficulty and increased uncertainty. It is possible to argue that the high-uncertainty condition also makes the target discrimination more difficult. Any observed differences in cueing effects between the high- and low-uncertainty conditions could then be attributed to increased difficulty and not uncertainty. Thus, we included a condition in which the uncertainty was low, but the difficulty in discriminating the target was high. Another major change compared to Shiu and Pashler (1994) was that we used cues that were nonpredictive of the target location (25% valid vs. 75% in Shiu and Pashler) to make them entirely task-irrelevant since our objective was to examine if curiosity-inducing uncertainty makes people more susceptible to task-irrelevant information.

In all the blocks, d primes were calculated based on hit rates and false alarm rates. Response times were also analyzed. We expected to replicate the main findings of Shiu and Pashler (1994) and observe greater cueing effects in the four-masks condition compared to the single-mask (easy) condition. This is because we expected the presentation of four masks to induce high uncertainty compared to the single mask trials. This increase in uncertainty would presumably lead to the induction of higher curiosity in the four-masks condition. Given our hypothesis that curiosity leads to greater processing of task-irrelevant information, we predicted that the participants would pay greater attention to the task-irrelevant peripheral cues in the four-masks condition, resulting in greater cueing effects. We also expected the overall error percentage to be the highest in the low uncertainty difficult condition.

### Method

#### Participants

The sample size was determined based on the sample size in Shiu and Pashler (1994) using a power analysis (“pwr” package in R). There were 12 participants in Shiu and Pashler (1994, Experiment 1). Cohen’s standardized difference score (*d*_*z*_; Cohen, 1988) estimated using the reported paired-sample *F* test values and sample sizes was 1.01. The calculations were based on results reflecting differences between cue valid and invalid trials. The power analysis yielded a sample size of 10 for a desired power of 0.8 with the confidence level set to 0.05. We selected a sample size that was much larger than this as we had an additional experimental condition (“low uncertainty difficult”). The participants were recruited through Prolific.

Thirty-nine individuals (20 females, 19 males, *M*_age_ = 25 years, *SD* = 4) were recruited on Prolific. All participants were healthy adults with no known neurological impairments in the age range of 19–35 years. Participants took part in the study in exchange for about €5. Informed consent was obtained from the participants. The experimental procedure was approved by the Ethics Committee of TU Dresden (BO-EK-8012020).

#### Apparatus

The experiment was designed using PsychoPy and administered online on Pavlovia. Participants were instructed to sit in a quiet room, keep their phones on silent mode/switched off, not listen to music or multitask in any way and minimize distraction during the experiment. Participants were asked to sit 60 cm away from their computer screens. The stimuli sizes and the distances between them were controlled through a calibration process commonly used in online experiments (Li et al., 2020; Morys-Carter, 2021). Participants were shown a rectangular box and asked to adjust the size of the box to match that of a standard debit/credit card. They were informed that the cards would not be used for any financial transaction. Visual angle subtended by the card at a distance of 60 cm was then calculated. Credit/debit cards are typically chosen as the reference because they are of a standard size across the world which makes it convenient to calculate a base pixel/degree value based on each participant’s screen dimensions. This value was used as a base unit for all stimuli sizes and distances. For instance, if an object had to be presented at 2° visual angle and if the base value was 50 px/° for a given participant’s screen, then the stimulus size was calculated to be (50 × 2) px.

#### Procedure

**Figure 1:**
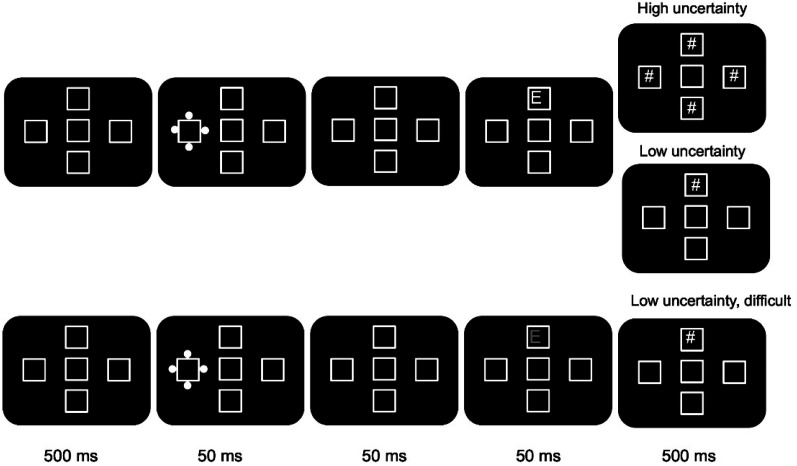
Sequence of events on a sample trial in Experiment 1. The mask display for all three experimental conditions is shown.

Following the calibration process, the main experimental procedure started with an instruction screen explaining the task to the participants. Each trial started with the presentation of four white square placeholders (2.5°) arranged in the shape of a plus sign and a central placeholder on a black background (Figure 1). After 500 ms, one of the four placeholders were surrounded by four white dots for 50 ms and functioned as the *abrupt-onset cue*. Following a brief gap of 50 ms, a single target letter (E or H, 2° height) was presented in white inside one of the four placeholders for 50 ms. Next, the symbol # (2° height) was presented for 500 ms to mask the visibility of the target. There were two types of trials: high uncertainty and low uncertainty. In the low-uncertainty trials, the #symbol was presented only at the location of the target letter. In the high-uncertainty condition, the # symbol was presented inside all four placeholders. Following the presentation of the mask (s), the participants were asked to judge if the target letter was E or H and press the corresponding key on the keyboard. The participants were instructed to pay full attention to the task and do their best. The trial terminated only after a response was registered.

There were 256 low-uncertainty and 256 high-uncertainty trials presented in two blocks. Additionally, there was a block of low-uncertainty difficult condition with 256 trials in which the task difficulty was increased. This was achieved by changing the color of the target letter to gray (No. 737380). This made the presentation of the target weaker and thereby reduced the contrast between the target letter and the background. The trial order and the block order were randomized during presentation.

At the beginning of the experiment, 24 practice trials were presented with feedback given only on incorrect responses. In the main experimental blocks, participants were allowed to take a self-paced break after each block of 64 trials. During the break, the total accuracy and mean response time in the preceding block were shown as feedback.

### Results

**Figure 2:**
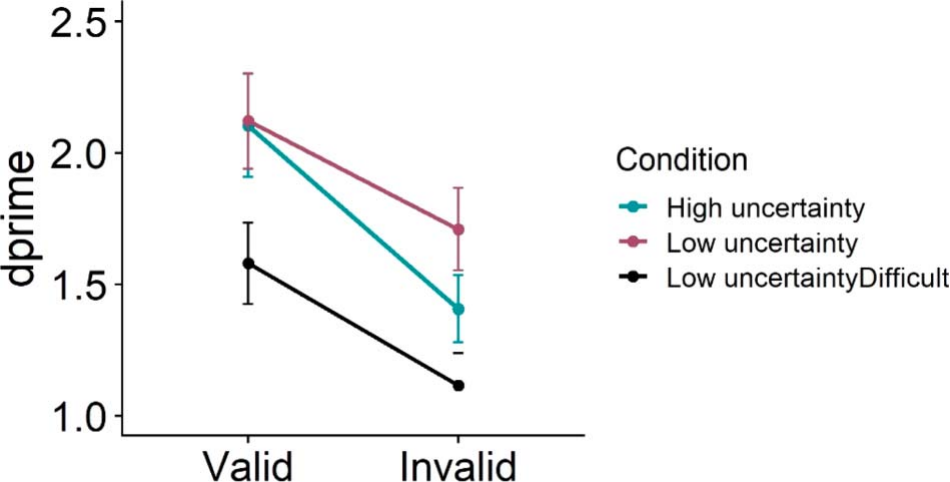
*d*′ values as a function of uncertainty conditions and cue validity (Experiment 1). Error bars represent between-participants error terms ±1 *SE*.

Data from two participants whose accuracy was below 55% were discarded from further analyses. Mean error rates and *d*′ were calculated (Figure 2, Table 1). “E” was considered a signal, and “H” was considered a noise. Note that these assignments are arbitrary and can be reversed. Hits constituted correct responses to “E,” and false alarms constituted incorrect responses to “H.” *d*′ score for each participant was calculated as the difference in *z*-transform values of hit rates and false alarms. Response times were also analyzed after discarding trials with incorrect responses (25%) and RTs faster than 100 ms or slower than 2,000 ms (3.6%).

**Table 1:** Mean RT and *d*′ values with *SD* values for all conditions in Experiment 1

Condition	*d*′		RT	
	Valid	Invalid	Valid	Invalid
High uncertainty	2.13 (1.19)	1.42 (0.77)	390 (129)	417 (132)
Low uncertainty	2.14 (1.08)	1.72 (0.96)	376 (143)	408 (150)
Low uncertainty – difficult	1.61 (0.93)	1.12 (0.74)	426 (172)	470 (197)

Repeated-measures ANOVA was performed on *d*′ values with condition (high uncertainty, low uncertainty, low uncertainty difficult) and validity (valid, invalid) as factors. There was a main effect of validity demonstrating the effectiveness of the cue, *F*(1, 36) = 44.14, *p* < .001, *pes* = .55. The *d*′ on valid trials was significantly higher compared to that on invalid trials. There was a main effect of condition, *F*(2, 72) = 9.21, *p* < .001, *pes* = .2, with lower *d*′ in the low-uncertainty difficult condition (1.22) compared to both low-uncertainty (1.81) and high-uncertainty conditions (1.54). This demonstrated that the low-uncertainty difficult condition did involve greater task difficulty. Importantly, a significant interaction between validity and condition was observed, *F*(2, 72) = 3.75, *p* = .028, *pes =* .09. The difference between valid and invalid trials was significant in all three conditions (*p* < .001). Pairwise comparisons showed that there was no difference in the valid trials between high- and low-uncertainty conditions (*p* = .946), but there was a significant difference between high-uncertainty invalid trials and low-uncertainty invalid trials (*p* = .023), suggesting greater cueing effects in the high-uncertainty condition. The *d*′ in the low-uncertainty difficult condition was significantly greater compared to the low-uncertainty condition for both valid (*p* = .002) and invalid trials (*p* < .001). Similarly, when comparing low-uncertainty difficult and high-uncertainty conditions, there was a significant difference on both valid (*p* = .009) and invalid (*p* = .02) trials. The cueing effect in the low-uncertainty difficult condition (0.42) was comparable to the cueing effect in the low-uncertainty condition (0.49). Further analyses were done to examine if the effects remained stable through the block. The trials in each condition were divided into four consecutive blocks, and Block (Block1, Block2, Block3, Block4) was entered as a factor in the repeated-measures ANOVA on *d*′ along with condition and validity. There was no main effect of Block, *F*(3, 108) = 0.51, *p* = .679, *pes =* .01. None of the 2-way or 3-way interactions with Block was significant either (*p* > .2).

Analyses of response times showed a significant main effect of validity, *F*(1, 36) = 27.07, *p* < .001, *pes* = .43, with faster response times on valid trials compared to invalid trials. There was only a marginal effect of condition, *F*(1.7, 60.3) = 3.43, *p* = .047, *pes* = .09, with no interaction between condition and validity, *F*(1.6, 56.7) = 1.59, *p* = .215, *pes* = .04.

### Discussion

We replicated the main findings of Shiu and Pashler (1994): More pronounced cueing effects were seen with higher uncertainty, suggesting that participants attended more to the abrupt-onset cues when the task involved higher uncertainty. Additionally, we showed that these effects cannot be merely attributed to the high-uncertainty condition, making it more difficult to find the target. In the low-uncertainty difficult condition, we kept the uncertainty low (single-mask) but increased the difficulty of finding the target. In this condition, there were overall more errors on both cue-valid and invalid trials, but there was no difference in cueing effect compared to the low-uncertainty condition. Thus, we can conclude that the increase in uncertainty due to the presentation of the four masks, and not merely the increase in difficulty, led to the observed increase in the cueing effects.

## Experiment 2

It is possible to question whether the findings of Experiment 1 could indeed be attributed to curiosity. Many previous studies on curiosity have elicited self-report ratings on participants’ level of curiosity. We wanted to investigate if participants in our study would report to be more curious in the high-uncertainty condition compared to the low-uncertainty conditions and if their curiosity ratings would positively correlate with cueing effects. The experimental design was exactly the same as in Experiment 1 with the addition of self-report ratings of curiosity on some trials (Gruber et al., 2014). This was done to examine 1) if the modulation of cueing effects as a function of uncertainty observed in Experiment 1 was reliable and 2) if those effects can indeed be attributed to the manipulation of curiosity. We expected the participants to report to be more curious on trials in the high-uncertainty condition compared to the low-uncertainty conditions. We also expected individuals reporting higher curiosity to show greater cueing effects.

### Method

#### Participants

Thirty-two individuals (7 females, *M*_age_ = 26.35 years, *SD* = 4.5) recruited on Prolific took part in the study in exchange for an average of €5.

#### Procedure

The procedure was exactly the same as in Experiment 1 with the following exception: A question regarding the participant’s curiosity level was asked on 25% of the trials in each of three conditions (64 trials in each condition). Participants were asked, “How curious are you about the letter that just appeared?” and they provided a response on a 6-point scale ranging from 1 (*not at all curious*) to 6 (*extremely curious*). Responses were made by pressing one of the six numeric keys (1, 2, 3, 4, 5, and 6). The curiosity question was asked after the participants had responded to the preceding target letter. There was no time constraint to respond to the curiosity question.

#### Results

**Figure 3:**
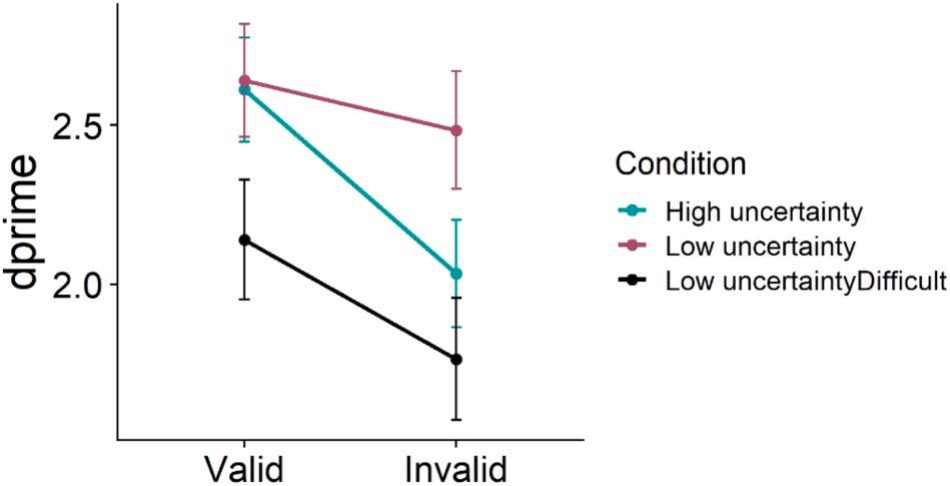
Condition-wise *d*′ values in Experiment 2. Error bars represent between-participants error terms ±1 *SE*.

Data from one participant were discarded from further analyses for having accuracy less than 55%. Trials with response times <100 ms and >2,000 ms (3%) and trials with incorrect responses were removed from RT analyses (16.4%). Repeated-measures ANOVA on *d*′ values showed a significant main effect of validity, *F*(1, 30) = 24, *p* < .001, *pes* = .44, and condition, *F*(2, 60) = 8.6, *p* < .001, *pes* = .22. Importantly, there was a significant interaction between condition and validity (see Figure 3, Table 2), as observed in Experiment 1, *F*(2, 60) = 5.53, *p* = .006, *pes* = .16. Pairwise comparisons showed that the interaction was driven by differences between high uncertainty invalid and low uncertainty invalid trials (*p* = .002). In contrast, the *d*′ value in the low uncertainty difficult condition was greater than the low-uncertainty condition on both valid (*p* = .009) and invalid (*p* < .001) trials, suggesting that there was an overall increase in difficulty in the low-uncertainty difficult condition compared to the low-uncertainty condition.

**Table 2:** Mean RT, *d*′ values and mean curiosity ratings with standard deviation values for all conditions in Experiment 2

Condition	*d*′		RT		Rating
	Valid	Invalid	Valid	Invalid	
High uncertainty	2.61 (0.91)	2.03 (0.94)	340 (146)	368 (147)	3.07 (1.5)
Low uncertainty	2.64 (0.98)	2.48 (1)	300 (111)	336 (136)	3.09 (1.5)
Low uncertainty – difficult	2.14 (1.05)	1.76 (1.07)	341 (155)	381 (171)	3.38 (1.3)

RT analyses showed a significant main effect of validity, *F*(1, 30) = 71.32, *p* < .001, *pes* = .7 and condition, *F*(2, 60) = 5, *p* = .01, *pes* = .14. There was no interaction between condition and validity, *F*(2, 60) = 1.67, *p* = .197, *pes* = .05.

Two participants’ data were discarded from the rating analyses because they pressed the same key (rating = 1) on ALL rating trials. Mean curiosity score was calculated for each condition. A repeated-measures ANOVA on condition and validity as factors revealed no main effect of condition, *F*(2, 56) = 1.52, *p* = .227, *pes* = .05. The average rating score did not differ between the three conditions (see Table 2 for means). There was neither a main effect of validity, *F*(1, 28) = 2.72, *p* = .11, *pes* = .09, nor an interaction between condition and validity, *F*(2, 56) = 2.07, *p* = .135, *pes* = .07.

**Figure 4:**
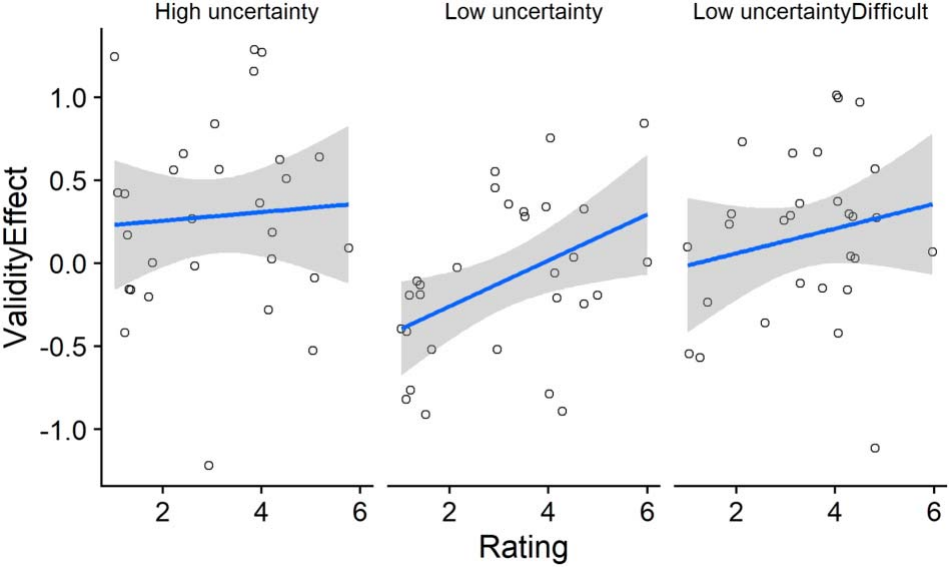
Correlation between validity effects (*d*′ on valid trials–*d*′ on invalid trials) and curiosity ratings for each of the uncertainty conditions.

We also examined if the performance on the cueing task can be predicted by the curiosity rating scores. To do this, a linear regression model was constructed using the lm() function in R. The difference in *d*′ values between valid and invalid trials (Validity Effects) was entered as the dependent variable. Trials within each condition were divided into four blocks of 16 trials each, and Block factor was created with four levels. This was done to examine if the cueing effects, the curiosity ratings, and any potential interaction between them remained stable over task blocks. The interaction between Rating score, Condition, and Block was entered into the model. There was no main effect of Block (*p* > .2), suggesting that the cueing effects remained stable over trials within each condition. There was no interaction between Block and Condition either (*p* > .1). Importantly, the interaction between Rating score and Condition was close to significance, *t* = 2, *p* = .045. Figure 4 suggests that this interaction is due to the positive correlation between Rating Score and Validity Effect in the easy low-uncertainty condition and the absence of correlations in the other two conditions. Individual correlations performed for each condition showed a significant positive correlation between rating Score and Validity Effect only in the low-uncertainty condition (*r* = 0.44, *p* = .016). There was no significant correlation in the other two conditions (high-uncertainty: *r* = 0.06, *p* = .74; low-uncertainty difficult: *r* = 0.19, *p* = .32).

### Discussion

We replicated the exact same pattern of results found in Experiment 1. Greater cueing effects were seen in the high-uncertainty condition compared to the low-uncertainty condition. The difference between the two conditions was driven primarily by differences on *d*′ on invalid trials, just like in Experiment 1. Overall accuracy was lower in the low-uncertainty difficult condition compared to the low-uncertainty condition, but there were no differences in the cueing effect indicating that target uncertainty and target difficulty impacted responses differently. Importantly, we elicited self-reported ratings on curiosity on a proportion of trials in each block. Contrary to our expectations, there were no differences in the mean curiosity ratings between the different uncertainty conditions.

To examine the role of individual differences in curiosity, we performed linear regression between the curiosity ratings and cueing effects in each condition. We observed a significant correlation between cueing effect and the rating score, but only in the low-uncertainty condition. That is, participants who reported to be more curious in the low-uncertainty block also showed greater cueing effects. Surprisingly, no such correlation was observed in the high-uncertainty block. It is worth noting that the analyses do not represent trial-wise correlations between curiosity ratings and cueing effects. Thus, we are not saying that trials on which people reported high curiosity also gave rise to higher cueing effects (in the low-uncertainty condition). Instead, the correlation was between mean curiosity rating in a condition (across 64 trials) and the mean cueing effect for that condition (across 256 trials). It is possible that the cueing effects in the high-uncertainty condition were already at ceiling for most participants and thus were not susceptible to variations in individual differences in self-reported curiosity. This seems a plausible explanation as it is unlikely that curiosity played a role ONLY in the low-uncertainty condition but not in the high-uncertainty condition. However, this still does not explain why such a correlation was not observed in the low-uncertainty difficult condition.

## General Discussion

In two experiments, we examined whether stimulus uncertainty modulates cueing effects in a spatial orienting task. We found the effect of spatial uncertainty in Experiments 1 and 2, where the presence of the masks made the target location uncertain. The effects of increasing uncertainty were also different from the effects of increasing task difficulty in Experiments 1 and 2. The difference between cue-valid and invalid trials was greater in the high-uncertainty condition than in the low-uncertainty condition. In the low-uncertainty difficult condition, however, the error percentage was higher on both cue-valid and invalid trials compared to the other low-uncertainty condition. Thus, while difficulty led to an overall increase in the percentage of errors, high uncertainty led to differences in the cueing effect (difference between cue-valid and invalid trials).

The results from the analyses of the self-report ratings in Experiment 2 were surprisingly inconclusive. Contrary to our expectations, there was no difference in the curiosity ratings between the high- and low-uncertainty conditions. It is possible that we would have seen a stronger effect of the uncertainty manipulation on the curiosity ratings if participants had been asked to rate their curiosity about the location of the target specifically (instead of being asked to rate their curiosity “about the letter that just appeared”). This method of eliciting self-report of curiosity has been successfully used in many previous studies (e.g., Gruber et al., 2014; Padulo et al., 2022). However, it is worth noting that in most of these studies curiosity was induced using more direct methods, such as trivia questions. It is possible that self-report of curiosity works well in such cases, whereas the curiosity triggered in our study, if at all, is not amenable to explicit self-report. Other indirect measures of subjective uncertainty and curiosity might be more useful. For instance, Nicki (1970, Experiment 3) showed a series of clear, lightly blurred, moderately blurred, and heavily blurred slides and asked participants to guess the identity of the objects. Subjective uncertainty was calculated as a function of the number of guesses made and the participant’s estimation of how certain they were of each guess. Such a measure could be better suited to tap into the kind of uncertainty present in our task.

Given this, it is possible to question if the uncertainty manipulation in the current study indeed induced curiosity. We relied on past studies on uncertainty and the theoretical framework on curiosity to assume the link between uncertainty and curiosity. But, we agree that we may not have been able to successfully provide empirical evidence connecting the two within this study. There was a significant correlation between the curiosity ratings and the cueing effects but only in the low-uncertainty condition, indicating that people who are highly curious also display larger cueing effects. The absence of such a correlation in the other two conditions is puzzling but the presence of the correlation in the low-uncertainty condition does seem to suggest that curiosity as a personality trait could play a role in modulating attention control. In line with this, it has already been seen that trait curiosity reliably predicts participants’ eye movements during scene viewing (Risko et al., 2012). Finding evidence for a direct causal role of curiosity in attention control would involve having a more effective within-subject manipulation of curiosity.

The design for Experiments 1 and 2 was borrowed from Shiu and Pashler (1994), and the results partly replicated the overall findings from Shiu and Pashler (1994) except in the single-mask (low-uncertainty) condition. The cueing effects in this condition were negligible in Shiu and Pashler (1994), whereas we observed significant cueing effects in the low-uncertainty condition in both Experiments 1 and 2. This can be explained through the curiosity-based arguments because we expected to observe cueing effects in all conditions and only predicted a magnitude difference between the different conditions. However, it is possible the uncertainty manipulation failed to induce curiosity in which case there must be other explanations for (1) the presence of cueing effects in the single-mask condition and (2) the reduction in its magnitude compared to the four-masks condition. A major difference between our study and that of Shiu and Pashler (1994) was that the cues in our study had 25% validity and were completely nonpredictive of the target location, whereas in Shiu and Pashler (1994) the cues were 75% valid making them predictive of the target location.

It is possible spatial cues allow exclusion of decision noise (leading to no cueing effects in Shiu and Pashler, Experiment 1) only when they are predictive of the target location. Thus, when the cues are nonpredictive (as in our study), cues do not lead to noise reduction as efficiently giving rise to cueing effects. Shiu and Pashler (1994) state that “With a blank field and a single target followed by a single mask, exclusion cannot help performance, so the validity of the precues does not matter. (p. 13).” This potentially suggests that they would expect no cueing effects even with nonpredictive cues. If so, our findings call their interpretation into question. Alternatively, it is possible that the single-mask condition makes participants strategically ignore the cues because the mask is a more reliable indicator of the target location resulting in a marginal (current study) or no effect (Shiu and Pashler) of the cues. Furthermore, Shiu and Pashler (1994) report only errors but not RT, and it is possible different effects of uncertainty are seen when both RT and Accuracy are considered (Prinzmetal et al., 2005).

Curiosity has traditionally been studied within personality psychology mostly as an individual trait (Litman & Silvia, 2006; Loewenstein, 1994). Recent research has shown that there may be only a partial overlap between the trait conceptualization of curiosity and curiosity seen as a cognitive state that drives information seeking (Jach et al., 2023). Furthermore, robust and reliable cognitive tasks, such as the cueing paradigm, are not necessarily well-suited to tap into individual differences (Hedge et al., 2018). Given this, seeking correlations between self-reports and performance on a cognitive task might not be enough or useful. Experimentally inducing a complex cognitive state such as curiosity in a laboratory setting is not easy either. Previous research along these lines has used trivia questions or lottery tasks to study the influence of curiosity on learning or memory, for instance (Galli et al., 2018; Gruber et al., 2014; Kidd & Hayden, 2015; Van Lieshout et al., 2018). Incorporating these approaches into classic cognitive paradigms such as spatial cueing or flanker task is not straightforward and makes assumptions about the far transfer of cognitive states. In sum, we acknowledge that we cannot conclude that the uncertainty manipulation indeed led to changes in curiosity. There is a great need for developing novel methods that unequivocally induce a cognitive state of curiosity and provide a framework for studying its relationship with other cognitive functions such as attention and cognitive control.

In conclusion, it is important to consider that human beings often entertain a variety of goals and motivations that do not disappear as soon as they start a laboratory experiment. Instances of task-irrelevant processing could be the result of these factors and not necessarily demonstrate a breakdown of intentional control. This is also in line with the metacontrol framework which proposes that individuals exhibit two styles of processing: persistence and flexibility (Hommel, 2015). The choice of the processing style depends on the nature of the task, individual differences in motivation, personality, and cultural background, among other factors. A persistent metacontrol state facilitates the processing of information consistent with the current task goal, whereas a flexible metacontrol state opens up the action system to a wide range of information that may satisfy other, concurrently active goals. For instance, it has been shown that focused-attention meditation induces a persistent style of information processing, whereas open-monitoring meditation induces a flexible style (Hommel & Colzato, 2017). We propose that curiosity, similarly, could make individuals adopt a flexible metacontrol style, which encourages processing of seemingly task-irrelevant information. Establishing such a link between curiosity, metacontrol, and other cognitive functions through further experiments with a compelling manipulation of curiosity is necessary to provide a comprehensive outlook to cognition.

## Notes

**Authorship:** Seema Prasad, conceptualization, methodology, software, analyses, writing – original draft; Bernhard Hommel, conceptualization, writing – review and editing.

**Open Data:** The raw data analyzed and presented in the manuscript can be found here: https://osf.io/gqn7t/?view_only=101d216bc9064d009b409525397d20a7 (Prasad, 2024).

## References

[c1] Awh, E., Belopolsky, A. V., & Theeuwes, J. (2012). Top-down versus bottom-up attentional control: A failed theoretical dichotomy. *Trends in Cognitive Sciences*, *16*(8), 437–443. 10.1016/j.tics.2012.06.010 22795563 PMC3426354

[c2] Bahrick, L. E., Walker, A. S., & Neisser, U. (1981). Selective looking by infants. *Cognitive Psychology*, *13*(3), 377–390. 10.1016/0010-0285(81)90014-1 7237992

[c3] Berlyne, D. E. (1960). *Conflict, arousal, and curiosity**.* McGraw-Hill Book Company. 10.1037/11164-000

[c4] Bromberg-Martin, E. S., & Hikosaka, O. (2009). Midbrain Dopamine neurons signal preference for advance information about upcoming rewards. *Neuron*, *63*(1), 119–126. 10.1016/j.neuron.2009.06.009 19607797 PMC2723053

[c5] Cohen, J. (1988). *Statistical power analysis for the behavioral sciences* (2nd ed.). Lawrence Erlbaum Associates.

[c7] Galli, G., Sirota, M., Gruber, M. J., Ivanof, B. E., Ganesh, J., Materassi, M., Thorpe, A., Loaiza, V., Cappelletti, M., & Craik, F. I. M. (2018). Learning facts during aging: The benefits of curiosity. *Experimental Aging Research*, *44*(4), 311–328. 10.1080/0361073X.2018.1477355 29787342

[c8] Gottlieb, J. (2023). Emerging principles of attention and information demand. *Current Directions in Psychological Science*, *32*(2), 152–159. 10.1177/09637214221142778

[c9] Gottlieb, J., Cohanpour, M., Li, Y., Singletary, N., & Zabeh, E. (2020). Curiosity, information demand and attentional priority. *Current Opinion in Behavioral Sciences*, *35*, 83–91. 10.1016/j.cobeha.2020.07.016

[c10] Gruber, M. J., Gelman, B. D., & Ranganath, C. (2014). States of curiosity modulate hippocampus-dependent learning via the dopaminergic circuit. *Neuron*, *84*(2), 486–496. 10.1016/j.neuron.2014.08.060 25284006 PMC4252494

[c11] Hedge, C., Powell, G., & Sumner, P. (2018). The reliability paradox: Why robust cognitive tasks do not produce reliable individual differences. *Behavior Research Methods*, *50*(3), 1166–1186. 10.3758/s13428-017-0935-1 28726177 PMC5990556

[c12] Hommel, B. (2015). Between persistence and flexibility: The Yin and Yang of action control. In A. J. Elliot (Ed.), *Advances in motivation science* (Vol. 2, pp. 33–67). Elsevier.

[c13] Hommel, B. (2022). GOALIATH: A theory of goal-directed behavior. *Psychological Research*, *86*(4), 1054–1077. 10.1007/s00426-021-01563-w 34324040 PMC9090680

[c14] Hommel, B., & Colzato, L. S. (2017). Meditation and metacontrol. *Journal of Cognitive Enhancement*, *1*(2), 115–121. 10.1007/s41465-017-0017-4 32226918 PMC7089713

[c15] Hommel, B., & Wiers, R. W. (2017). Towards a unitary approach to human action control. *Trends in Cognitive Sciences*, *21*(12), 940–949. 10.1016/j.tics.2017.09.009 29150000

[c16] Hunter, M. A., & Ames, E. W. (1988). A multifactor model of infant preferences for novel and familiar stimuli. *Advances in Infancy Research*, *5*, 69–95.

[c17] Jach, H., Cools, R., Frisvold, A., Grubb, M. A., Hartley, C. A., Hartmann, J., Hunter, L., Jia, R., De Lange, F., Larisch, R., Lavelle-Hill, R. E., Levy, I., Li, Y., Van Lieshout, L., Nussenbaum, K., Ravaioli, S., Wang, S., Wilson, R. C., Woodford, M., ... Gottlieb, J. (2023). *Curiosity in cognitive science and personality psychology: Individual differences in information demand have a low dimensional structure that is predicted by personality traits* [Preprint]. PsyArXiv. 10.31234/osf.io/aj3rp PMC11551435 39467138

[c19] Kidd, C., & Hayden, B. Y. (2015). The psychology and neuroscience of curiosity. *Neuron*, *88*(3), 449–460. 10.1016/j.neuron.2015.09.010 26539887 PMC4635443

[c20] Li, Q., Joo, S. J., Yeatman, J. D., & Reinecke, K. (2020). Controlling for participants’ viewing distance in large-scale, psychophysical online experiments using a virtual chinrest. *Scientific Reports*, *10*(1), Article 904. 10.1038/s41598-019-57204-1 31969579 PMC6976612

[c101] Li, Q., Joo, S. J., Yeatman, J. D., & Reinecke, K. (2020). Controlling for participants’ viewing distance in large-scale, psychophysical online experiments using a virtual chinrest. *Scientific Reports*, *10*(1), Article 904. 10.1038/s41598-019-57204-1 31969579 PMC6976612

[c21] Litman, J. A., & Silvia, P. J. (2006). The latent structure of trait curiosity: Evidence for interest and deprivation curiosity dimensions. *Journal of Personality Assessment*, *86*(3), 318–328. 10.1207/s15327752jpa8603_07 16740115

[c22] Loewenstein, G. (1994). The psychology of curiosity: A review and reinterpretation. *Psychological Bulletin*, *116*(1), 75–98. 10.1037/0033-2909.116.1.75

[c23] Morys-Carter, W. L. (2021, May 18). *ScreenScale* [Computer software]. Pavlovia. 10.17605/OSF.IO/8FHQK

[c24] Nicki, R. M. (1970). The reinforcing effect of uncertainty reduction on a human operant. *Canadian Journal of Psychology/Revue canadienne de psychologie*, *24*(6), 389–400. 10.1037/h0082875

[c25] Padulo, C., Marascia, E., Conte, N., Passarello, N., Mandolesi, L., & Fairfield, B. (2022). Curiosity Killed the cat but not memory: Enhanced performance in high-curiosity states. *Brain Sciences*, *12*(7), Article 846. 10.3390/brainsci12070846 35884653 PMC9313209

[c26] Posner, M. I. (1980). Orienting of attention. *Quarterly Journal of Experimental Psychology*, *32*(1), 3–25. 10.1080/17470218.2014.937446 7367577

[c122] Prasad, S. (2024). *Curiosity and attention control* [Data]. https://osf.io/gqn7t/?view_only=101d216bc9064d009b409525397d20a7 10.1027/1618-3169/a000608 PMC11601269 38682782

[c27] Prinzmetal, W., McCool, C., & Park, S. (2005). Attention: Reaction time and accuracy reveal different mechanisms. *Journal of Experimental Psychology: General*, *134*(1), 73–92. 10.1037/0096-3445.134.1.73 15702964

[c120] Risko, E. F., Anderson, N. C., Lanthier, S., & Kingstone, A. (2012). Curious eyes: Individual differences in personality predict eye movement behavior in scene-viewing. *Cognition*, *122*(1), 86–90. 21983424 10.1016/j.cognition.2011.08.014

[c29] Shiu, L. P., & Pashler, H. (1994). Negligible effect of spatial precuing on identification of single digits. *Journal of Experimental Psychology: Human Perception and Performance*, *20*(5), 1037–1054. 10.1037/0096-1523.20.5.1037

[c30] Snowden, R. J., Willey, J., & Muir, J. L. (2001). Visuospatial attention: The role of target contrast and task difficulty when assessing the effects of cues. *Perception*, *30*(8), 983–991. 10.1037/0096-1523.20.5.1037 11578083

[c100] Snowden, R. J., Willey, J., & Muir, J. L. (2001). Visuospatial attention: The role of target contrast and task difficulty when assessing the effects of cues. *Perception*, *30*(8), 983–991. 10.1068/p3068 11578083

[c31] van Lieshout, L. L. F., de Lange, F. P., & Cools, R. (2020). Why so curious? Quantifying mechanisms of information seeking. *Current Opinion in Behavioral Sciences*, *35*, 112–117. 10.1016/j.cobeha.2020.08.005

[c32] Van Lieshout, L. L. F., Traast, I. J., De Lange, F. P., & Cools, R. (2021). Curiosity or savouring? Information seeking is modulated by both uncertainty and valence. *PLoS ONE*, *16*(9), Article e0257011. 10.1371/journal.pone.0257011 34559816 PMC8462690

[c33] Van Lieshout, L. L. F., Vandenbroucke, A. R. E., Müller, N. C. J., Cools, R., & De Lange, F. P. (2018). Induction and Relief of curiosity elicit parietal and frontal activity. *The Journal of Neuroscience*, *38*(10), 2579–2588. 10.1523/JNEUROSCI.2816-17.2018 29439166 PMC6705901

[c34] Wang, M. Z., & Hayden, B. Y. (2019). Monkeys are curious about counterfactual outcomes. *Cognition*, *189*, 1–10. 10.1016/j.cognition.2019.03.009 30889493 PMC8029581

